# The Multifaceted Roles of Lamins in Lung Cancer and DNA Damage Response

**DOI:** 10.3390/cancers15235501

**Published:** 2023-11-21

**Authors:** Janina Janetzko, Sebastian Oeck, Alexander Schramm

**Affiliations:** Department of Medical Oncology, West German Cancer Center, University Hospital Essen, University of Duisburg-Essen, 45147 Essen, Germany; janina.janetzko@uk-essen.de (J.J.); alexander.schramm@uk-essen.de (A.S.)

**Keywords:** lamins, lung cancer, lamin composition, DNA repair, kinases

## Abstract

**Simple Summary:**

Lamins are a family of nuclear proteins consisting of A- and B-type lamins. As they are integral parts of the nuclear lamina, it was generally accepted that they have mainly structural functions. However, recent reports suggest a broader role for lamins in the context of cancer development and progression. Both the ratio of the different lamin subtypes as well as post-translational modifications can affect different cell states and processes including cellular stiffness, chromatin condensation, and regulation of the cell cycle. Here, we discuss the recent developments in lamin physiology and pathophysiology with a special focus on sometimes conflicting reports that describe the role of lamins in the context of lung cancer and the DNA damage response.

**Abstract:**

Emerging evidence suggests that lamin functions are not limited to maintaining the structural integrity of the nucleus in eukaryotic cells but that these functions affect many facets of cancer biology. An increasing number of reports suggest that adaptive changes in the lamin subtype composition within the nuclear lamina could affect essential features of cancer development and aggressiveness. These include regulation of cellular stiffness and mobility as well as epithelial-to-mesenchymal transition (EMT), all of which directly impact the metastatic properties of cancer cells. Additionally, insights from studies on the physiological functions of lamins suggest that cancer cells could hijack the ability of lamins to modify chromatin accessibility, cell cycle regulation, and DNA damage response. Here, we present a comprehensive overview of the role of lamins in lung cancer and DNA damage response, which is commonly evoked by lung cancer therapies. Collectively, this information should help better understand the sometimes-conflicting reports on lamin functions in lung cancer as well as in other cancer types.

## 1. Introduction

Lamins are type V intermediate filament proteins that are best known for their scaffolding function in the nucleus of eukaryotic cells [1,2]. Lamins are encoded by the *LMNA*, *LMNB1*, and *LMNB2* genes, giving rise to seven known lamin variants due to alternative splicing [3]. In physiological settings, lamins play important roles in maintaining the integrity of the nuclear envelope, regulating DNA replication and transcription, and organizing the chromatin structure [4,5]. Germline alterations in the lamin-encoding genes give rise to a multitude of disorders such as disturbed fat and skeletal homeostasis and syndromes that are summarized as laminopathies. These syndromes include cardiomyopathies, muscular dystrophies, and premature-aging-like syndromes such as the Hutchinson–Gilford progeria syndrome (HGPS). HGPS is caused by abnormal splicing of prelamin A, resulting in a shortened isoform that is referred to as lamin AΔ50, AΔ150, or progerin ([6], also reviewed in [7]). Lamin B-related diseases include lipodystrophy and brain disorders such as adult-onset autosomal dominant leukodystrophy (ADLD). While laminopathies are rare diseases, the underlying mutations provide insights into the function and organization of lamin proteins. Despite the fact that somatic mutations in the *LMNA* gene can occur in a variety of tumor types, these mutations are rare, and the mutation frequency does not exceed 3% in all cancer types analyzed by TCGA projects (Figure S1). In common cancers such as lung, prostate, and pancreatic tumors, lamin mutations occur in about 1% of cases, while the mutation rate was reportedly to be highest for *LMNB2* in esophageal carcinomas (2.8%) [8]. However, given the overall low mutation rates of *LMNA* and *LMNB* genes, differences in mutation frequencies appear to be marginal (Figure S1). This, in turn, does not suggest a causal role for the mutation of a specific lamin-encoding gene in tissue-specific cancer development. By contrast, changes in the expression and localization of lamins have been frequently reported across cancer types (Table 1), indicating that lamins could contribute to the progression rather than to the onset of the disease. Changes in lamin expression and distribution will affect the integrity of the nuclear envelope, possibly contributing to chromosomal and genomic instability [9,10], which are hallmark features of cancer. Consequently, aberrant localization and expression, especially of lamin B proteins, have been associated with cancer development, aggressiveness, and metastasis (reviewed in [11]). 

In this review, we first describe the physiological roles of lamins and put this into the context of the involvement of lamins governing genomic stability. We next analyze the differences of lamin expression reported in several types of cancer. Our main topic, lung cancer, is a model disease to further study lamins in the context of DNA damage response (DDR), chromatin organization, and their influence on EMT, as platinum-based chemotherapy and radiation, two mainstays in the therapy of lung cancer, both invoke DDR and DNA repair pathways. Although the interplay between these therapy-induced damage responses and changes in lamin expression or composition is not well understood, it might add to a better understanding of conflicting reports on lamins across cancer types. 

## 2. Physiological Role of Lamins and Their Regulation during Cell Cycle and Cell Division

**Figure 1:**
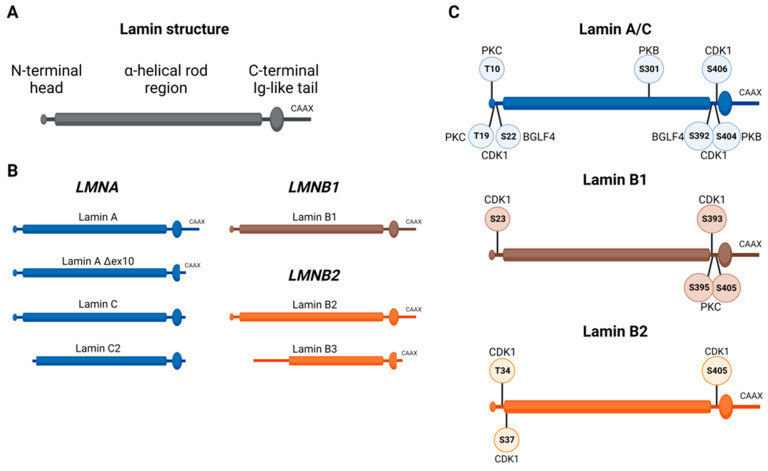
Lamin family members and prominent phosphorylation sites. (**A**) Schematic summary of the structure common to all lamin subtypes. (**B**) Overview of known lamin splice variants encoded by *LMNA* (blue), *LMNB1* (brown), and *LMNB2* (orange). (**C**) Major lamin phosphorylation sites targeted by oncogenes including CDK1 and AKT (PKB).

The nuclear lamina is a filamentous structure that determines the size, stability, and shape of the nucleus in eukaryotes [1]. The lamina consists mainly of type V intermediate filament proteins including A-type and B-type lamins [3]. A-type lamins are all encoded by the *LMNA* gene and can be divided into the four known alternative splice variants of lamin A, lamin C, lamin AΔex10, and lamin C2 [2,3,12]. B-type lamins are separately encoded by *LMNB1* and *LMNB*2, which are translated into lamin B1 and lamin B2, respectively. A splice variant, lamin B3, is expressed from the *LMNB2* gene using an alternative spermatid-specific promotor [13,14] (Figure 1B). A- and B-type lamins share the same domain structure consisting of an N-terminal head domain, a central α-helical rod domain including four coiled-coil segments, and a C-terminal IG-like tail domain (reviewed in [15]) (Figure 1A). Lamins form coiled-coil head-to-head dimers further connect to longitudinally linked head-to-tail polymers, which align laterally in an antiparallel manner to form tetrameric filaments [16]. Moreover, these tetrameric filaments are formed individually for A- and B-type lamins, but they build meshworks interacting with the nuclear envelope [17,18].

The phosphorylation of lamin domains prevents polymerization and thus impacts the lamin interactome (reviewed in [19]). While lamins contribute to nuclear integrity and stability, concerted lamin phosphorylation leading to depolymerization of the nuclear lamin structure and subsequent nuclear envelope breakdown is a prerequisite for cell division. Multiple phosphorylation sites within lamins are targeted by different kinases, including the cyclin-dependent kinase 1 (CDK1) [20], protein kinase A (PKA) [21], AKT (PKB) [22], protein kinase C (PKC) [21], mitogen-activated protein kinase MAPK [23] and casein kinase II [24] (Figure 1C).

During the interphase, when the nuclear lamina is attached to the nuclear envelope, lamin A/C interacts with emerin, an inner nuclear membrane protein [25], and both cooperate with nuclear myosin 1 (NM1) and actin to modulate chromatin organization. Loss of lamin A/C and emerin affects NM1 activity, which results in enhanced chromatin mobility, indicating that these interactions are important for maintaining chromosome positions during the interphase [26]. In prophase, DNA becomes condensed, and the nuclear envelope starts to break down (NEBD). During NEBD, the nuclear lamina becomes depolymerized and nuclear pore complexes (NPCs) dissociate, thus disrupting their interaction with chromatin. AKT and CDK1 were both reported to induce NEBD [20,27,28]. AKT was found to phosphorylate lamin A/C at Ser301 and Ser404 in C2C12 mouse myoblast cells [29,30] (Figure 1C). Bertacchini et al. [30] showed that the phosphorylation of prelamin A and lamin A/C by AKT marks both for degradation. Additionally, they reported AKT-mediated transcriptional control of *LMNA*, as prelamin A expression was downregulated in AKT-overexpressing cells. Complimentary results were obtained upon AKT knockdown and inhibition, which resulted in *LMNA* upregulation [31]. AKT activity peaks in the G2/M phase and drops right after mitosis [28], which recapitulates the coordinated and complex interactions underlying NEBD. Similarly, CDK1 is a prominent kinase phosphorylating lamins and NPC proteins to allow NEBD once CKD1 is activated by cyclin B1 [20,27]. Jeong et al. [32] showed that CDK1 activity is sufficient to disrupt the interaction of two parallel lamin dimers, which can be attenuated by casein kinase 1 (CK1) and glycogen synthase kinase-3 beta (GSK3β). The CDK1 complex targets several sites within different lamins, including lamin A/C (Thr19, Ser22, Ser392 and Ser404), lamin B1 (Ser23 and Ser393), and lamin B2 (Thr34, Ser37 and Ser405) (Figure 1C). All of these phosphorylation events contribute to lamin depolymerization and NEBD before the mitotic spindle assembles [20,33,34,35]. Upon NEBD, NPC components are distributed between the endoplasmic reticulum (ER) and cytoplasm [36]. Colocalization of lamin A/C and emerin is gradually lost in prometaphase, leading to the diffusion of lamins throughout the cell. By contrast, emerin remains detectable in the cytoplasm throughout mitosis, indicating a function of emerin for nuclear reassembly [37]. During cell division, B-lamins but not lamin A/C were reported to associate with the mitotic spindle. Using siRNA-induced depletion of lamin B1 or B2, Tsai et al. [38] showed increased spindle defects indicated by a significant increase in unfocused poles and/or a lack of chromosome alignment compared to control cells. These results indicate a functional role of B-type lamins during spindle assembly. After the alignment of chromosomes during metaphase, the NE starts to reassemble during ana- and telophase. With a gradual decrease of CDK1/CyclinB1 levels, the dephosphorylation of lamins by distinct phosphatases allows for re-polymerization and lamin A/C association with and around chromatin ([37], also reviewed in [39]). These observations indicate distinct roles of the different lamin types during cell division.

## 3. Contribution of Lamins to the Maintenance of Genomic Stability and Their Role in the DNA Damage Response

Aberrant DNA damage response (DDR) and evasion of senescence are hallmarks of cancer development and progression. DDR pathways include damage sensors, signal transducers, and effectors. Their deregulation contributes to genomic instability, which is associated with cancer onset and progression [40]. Cancer cells evade senescence either by activation of the enzyme telomerase (hTERT), which adds telomeric repeats to telomeres, or by utilizing the alternative lengthening of the telomer (ALT) pathway, which is based on homologous-directed repair (HDR) (reviewed in [41]). Tumor-specific strategies for stabilizing telomeres involve activation of hTERT by genomic rearrangements, mutational activation by point mutations, DNA amplification, and transcript fusions [42]. By contrast, the contribution of structural elements to telomere stability in cancer cells is not entirely understood. Crabbe et al. [43] reported the association of telomeres at the nuclear envelope during nuclear reassembly. Using an immuno-FISH approach, Pennarun et al. [44] demonstrated that DNA damage foci at telomeres were upregulated in lamin B1-overexpressing cells. Moreover, the integrity of the shelterin complex, which is essential for telomere protection, is affected by lamin B1 levels. Overexpression of lamin B1 induced mis-localization of the shelterin-complex members TRF2 and RAP1, subsequently leading to telomere instability [44]. This is functionally relevant, as a loss of TRF2 or RAP1 function increased the DDR and senescence [45,46,47]. However, the contribution of non-genetic stabilization of telomeres to telomere maintenance in cancer cells requires further investigation.

Another crucial factor that mediates proper DDR is the tumor suppressor p53 encoded by the *TP53* gene (reviewed in [48]). Upon DNA damage, p53 becomes phosphorylated by the ataxia-telangiectasia mutated (ATM) kinase, and the checkpoint kinase 2 (Chk2) releases p53 from the mouse double minute 2 homolog (MDM2), which targets p53 for proteasomal degradation. Active p53 induces, among others, the expression of the cyclin-dependent kinase inhibitors p21 and p16, causing the cell cycle arrest or cellular senescence, depending on the context (reviewed in [49]). Interestingly, Yoon et al. [50] reported a direct interaction between p53 and lamin A/C, but not lamin B, using in vitro binding assays. Moreover, increased p53 levels preferentially stabilize lamin A/C, which promotes nuclear deformation and induction of p16 [50]. In the same manuscript, the authors speculated that lamin A/C stabilization might be sufficient to induce senescence [50]. By contrast, Chuang et al. [51] reported that inactivation of the focal adhesion kinase (FAK) leads to upregulation of p53, which induced cellular senescence while downregulating lamin A/C. Additionally, they suggested FAK-mediated regulation of lamin A/C expression as a means to stabilize the nuclear organization and to activate anti-senescence programs [51]. However, Golubovskaya et al. [52] have shown that this interaction may be bi-directional, as p53 binds to the FAK promotor, thus inhibiting its activity. By analyzing the effect of lamin A/C depletion on the nuclear envelope and focal adhesions, Goelzer et al. [53] found upregulated FAK and AKT levels in *LMNA*-depleted cells and suggested this upregulation as compensation for cell softening upon lamin A/C deficiency. Together, these results indicate FAK as another important regulator in p53-mediated senescence through its interaction with lamin A/C and p53. At present, it remains unclear how these mutual interactions between FAK, p53, and lamin A/C are coordinated to regulate DDR and cellular senescence. 

In addition, lamin B1 was reported as a p53 target. Loss of p53 leads to upregulation of lamin B1 and other members of the nuclear lamina, including the nuclear pore complex 210 (Nup210). Both coding genes share genomic binding sites with p53, which was also functionally relevant in a pancreatic ductal adenocarcinoma cell line [54]. Consistent with these findings, Lämmerhirt et al. [55] reported that loss of lamin B1 induces senescence in melanoma cells, while Freund et al. [56] found that lamin B1 down-regulated upon the activation of p53 or pRB/p16^INK4a^. Following this observation, Onorati et al. [57] recently showed that different means to induce senescence, including replication exhaustion, expression of HRAS-V12, etoposide-mediated DNA damage, or ionizing irradiation, all resulted in decreased lamin B1 levels. These results indicate that p53, with or without the involvement of FAK, has opposing effects on the stabilization of lamin A/C and lamin B1, which thus may be an important switch regulating cellular senescence upon DNA damage.

The persistence of DNA double-strand breaks (DSBs), which are lethal when unrepaired, poses a dangerous threat to genomic integrity. Here, p53-binding protein 1, 53BP1 [58], is an important factor facilitating DSB repair, among others by protecting DNA ends from resection [59]. Structurally, the tudor and the ubiquitination-dependent recruitment (UDR) domains of 53BP1 enable its interaction with DSB-specific histone marks, H4K20me2, and ubiquitinated lysine 15 on H2A-type histones (H2A-K15ub) [60,61], thus establishing 53BP1 as a reader of DNA damage. Gibbs-Seymour et al. [62] described that lamin A/C specifically interacts with 53BP1s via the tudor domain. Here, irradiation-induced DNA DSBs resulted in a decreased interaction of lamin A/C and 53BP1 [62]. This interaction is functionally relevant, as lamin A/C depletion also resulted in a loss of the 53BP1 protein and concomitantly accumulated DNA damage [63]. Interestingly, proximity ligation assays also suggested a direct interaction between 53BP1 and lamin B1, which reportedly also stabilized the 53BP1–lamin A/C complex [64]. However, overexpression of lamin B1 resulted in impaired 53BP1 recruitment to DNA damage sites, decreased NHEJ, and thus resulted in the accumulation of unrepaired DNA DSB. Taken together, these results indicate distinct roles for lamin A/C and lamin B1 during DNA damage response and DNA double-strand repair that need further investigation to specify their function. 

Of note, mutant *LMNA^D300N^* predisposing to dilated cardiomyopathy in humans was shown to induce an aberrant DNA damage response involving ATM and p53, which could be partly rescued by p53 deletion in transgenic mice [65]. It is thus tempting to speculate that interfering with p53 and the DDR in laminopathies might ameliorate the pathophysiological consequences of lamin mutations. Additionally, Gonzalez-Suarez et al. [63] showed that interaction of lamin A/C with the DDR machinery is not restricted to its interaction with p53 pathway members, but can also involve telomeres. In *LMNA*^−/−^ mouse embryo fibroblasts (MEFs), telomeres were significantly shorter than in *LMNA*-wt MEFs, which correlated with a decrease in H4K20me3, a histone mark of telomeric heterochromatin. Additionally, loss of A-type lamins caused nuclear decompartmentalization of telomeres and hindered the ability to process dysfunctional telomeres [63]. These reports indicate that lamin A/C and B1 target distinct aspects of telomere stability, DDR, and senescence that determine their impact on pathophysiology.

## 4. Role of Lamins in Lung Cancer

Lung cancer is divided into two subtypes, namely the more frequently occurring non-small-cell lung cancers (NSCLC) and small-cell lung cancers (SCLC), which account for roughly 85% and 15% of all lung cancers, respectively. Depending on the subtype, varying levels of lamin A/C were detected in lung tumor cells. SCLC expresses *LMNA* at lower levels compared to NSCLC tumor tissues [66,67]. In pre-clinical models, conflicting results have been reported by Stefanello et al. [68], who found decreased lamin A/C levels in the lung adenocarcinoma (LUAD) cell line A549, while Rubporn et al. [69] reported *LMNA* overexpression in the same cell line. Furthermore, Hu et al. [70] reported that lamin A/C contributes to acquired resistance to erlotinib in *EGFR*-mutant NSCLC. They analyzed cytoskeletal changes and found increased nuclear deformation upon the modulation of *LMNA* expression. Based on these findings, they suggested that reduced *LMNA* expression contributes to higher nuclear plasticity. As for lamin A/C, different effects of lamin B1 on lung cancer biology were reported: Jia et al. [67] identified decreased lamin B1 expression as a lung cancer-promoting factor, whereas two reports by Li et al. [71] and Li et al. [72] suggested that lamin B1 overexpression correlated with advanced-stage NSCLC. Interestingly, lamin B2 expression levels were unanimously correlated with a higher clinical stage [73,74,75]; however, the mechanism underlying lamin B2 regulation still needs clarification. Taken together, dysregulation of the different lamin proteins is observed in lung cancer, but the impact derived thereof remains controversial. However, three eminent functions of lamins have been identified that seem to depend on the expression of the different lamin subtypes and that can be hijacked in lung cancer cells: 1. cell cycle regulation induced by receptor tyrosine kinase or protein kinase signaling that affects lamin polymerization by phosphorylation; 2. chromatin organization and compactness that can impact transcriptional activity; and 3. cellular stiffness and mobility that can be molecularly linked to lamin interaction with proteins regulating EMT. Beyond these central processes, lamins might also contribute to DNA damage response and repair pathways ([76], also reviewed in [77]).

### 4.1. Role of Lamin Dysregulation in DNA Damage Response, Cell Cycle Transition, and DNA Repair

The facts that nuclear integrity mediated by lamins controls cell fate and that rupture of the nuclear lamina is a catastrophic event causing programmed cell death are well acknowledged. However, in genomically unstable cancer cells, controlling functions of lamins can be overruled and hijacked. A prime example is the formation of lamin B1-containing micronuclei in lung cancer cells that form around missegregated chromosomes during mitotic exit, which in turn can contribute to genomic instability and even potentiate it [76]. Still, this structural function of lamins is only one side of the coin, as lamins can also interact with proteins that directly control cell cycle and proliferation.

Rubporn et al. [69] found that lamin A/C and several co-regulated proteins including KAP1 (TIF1β, TRIM28) were overexpressed in the A549 lung cancer cell line compared to MRC-5 normal lung fibroblast cells. Interestingly, Neumann-Staubitz et al. [33] identified KAP1 as an interactor of the Ig-domain of lamin C using genetically encoded crosslinkers. KAP1 is involved in cell survival after DNA damage and its phosphorylation by ATM, ATR, and DNA-PK recruits KAP1 to DNA strand breaks [78]. Furthermore, KAP1 is part of the transcription intermediary factor 1 (TIF1) sub-group, whose members possess a bromodomain (Bromo) and a plant homeodomain (PHD), which are common reader domains for the epigenetic code of histone post-translational modifications [79,80]. KAP1 was found to directly interact with MDM2, leading to the recruitment of the p53-HDAC1 complex that results in p53 inhibition and impaired apoptosis [81,82]. Consequently, the interaction of KAP1 with lamin C and its upregulation indicates a function of lamin A/C in cell survival in A549 cells, which needs to be verified in additional model systems.

In addition to lamin A/C, B-type lamins were reported to be deregulated in lung cancer. Immunohistology and biochemical analyses suggested that lamin B2 is upregulated in NSCLC and promotes aggressiveness by inducing or interacting with KLF16, MCM7, and G9a [73,74,75]. Here, expression of G9a depended on both lamin B2 and cyclin D1 expression, contributing to enhanced cell migration and cell cycle progression. Ma et al. [74] reported cell proliferation after overexpression of *LMNB2* and impaired G1/S cell cycle progression upon lamin B2 knockdown with concomitantly decreased levels of cyclin D1 and cyclin E1. Interestingly, cyclin B1 expression and G2/M phase transition seemed to be unaffected by lamin B2 levels, suggesting that lamin B2 specifically targets G1/S transition. However, overexpression of another lamin B2 interaction partner, KLF16, in H1299 and H1975 LUAD cell lines resulted in lamin B2 upregulation accompanied by enhanced proliferation and migration. Here, the fraction of cells in the G0/G1 phase decreased while G2/M cells were increased. Taken together, these results indicate that lamin B2 expression levels can impact cell cycle progression via different mechanisms.

### 4.2. Lamin Dysregulation and Chromatin Organization in Lung Cancer 

The interaction between chromatin and the nuclear lamina determines, at least in part, chromatin structure and accessibility [83]. At present, it is not known how the specificity of these interactions is regulated and how this physiological role of creating permissive or restrictive environments for gene transcription is hijacked in cancer cells. As described before, inactivation of FAK was reported to induce cellular senescence and p53 upregulation [51]. Recently, Chuang et al. [84] further investigated the mechanism of FAK-diminished senescence involving the enhancer of zeste homolog 2 (EZH2), which is a histone methyltransferase within the PRC2-repressor complex regulating H3K27 methylation and gene silencing [85,86]. Inhibition of EZH2 was previously reported to be involved in the onset of senescence in different cancer types such as triple-negative breast cancer or pancreatic cancer [87,88]. Furthermore, it was shown that FAK inhibition leads to senescence in lung cancer involving the downregulation of EZH2, suggesting a functional correlation of EZH2 and FAK in non-small cell lung cancer [84]. Interestingly, lamin B1 was reported to specifically interfere with the function of EZH2 [67]. By depleting lamin B1, Jia et al. [67] showed that chromatin-bound EZH2 was reduced concomitant with a decrease in H3K27me3. They suggested that lamin B1 is important for H3K27me3-mediated chromatin accessibility and transcriptional regulation [67]. These results and the aforementioned in vitro reports underscore that lamin A/C and lamin B1 could both contribute to the dysregulation of cellular senescence and epigenetic modifiers involved in cell division in lung cancer.

### 4.3. Impact of Lamin Dysregulation on Cellular Mobility, Plasticity, and EMT in Lung Cancer Cells and Lung Metastases

The plasticity and dynamic regulation of lamin proteins in lung cancer cells are highly debated and poorly understood. Hu et al. [70] investigated the consequences of downregulating *LMNA* in lung cancer cells on EMT by quantifying the ratio of E-cadherin and vimentin. In cells with reduced *LMNA* levels, E-cadherin was downregulated and vimentin was upregulated, indicating EMT accompanied by enhanced migration and invasion [70]. In a corresponding approach, Jia et al. [67] showed that depletion of lamin B1 in normal mouse lung epithelial cells induced the downregulation of E-cadherin and upregulation of the mesenchymal markers fibronectin, vimentin, and N-cadherin. Moreover, loss of lamin B1 led to upregulation of the proto-oncogene RET and its coreceptor GFRA1 together with upregulation of the MAP kinase p38. RET is a member of the receptor tyrosine kinase family and its activation stimulates several pathways, including the MAPK and PI3K/AKT signaling pathways (reviewed in [89]), which are involved in EMT (reviewed in [90]). Therefore, loss of lamin B1 could contribute to EMT via the activation of RET and its downstream function in the MAPK and PI3K/AKT pathways. On the contrary, Li et al. [71] reported that lamin B1 overexpression in LUAD was sufficient to activate the AKT/pAKT pathway. Depletion of lamin B1 slowed the tumor growth of A549 cells concomitant with decreased phosphorylation of AKT. They suggested that lamin B1 regulates cell proliferation in an AKT-dependent manner and that lamin B1 may function as an oncogene in LUAD. Interestingly, Pascual-Reguant et al. [91] showed that lamin B1 expression is essential for the acquisition of mesenchymal traits during EMT in the epithelial-like mouse cell line NMuMG. However, the role of lamin B1 in the context of EMT needs to be consolidated, as lamin B1 orchestrates signals from different pathways including AKT signaling, which could induce cell type-specific responses.

## 5. Discussion

**Figure 2:**
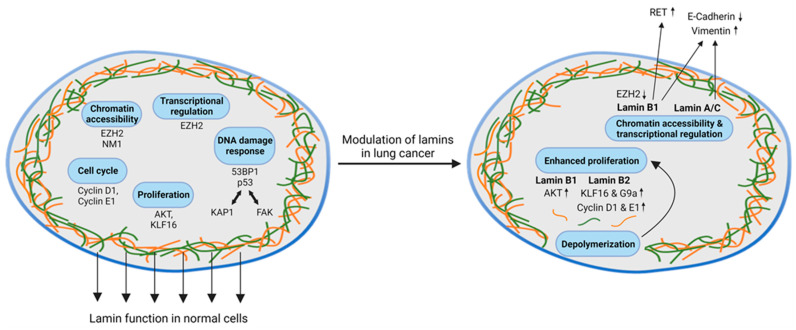
The multifaceted roles of lamins (orange, green) in maintaining cellular integrity and the DNA damage response in physiological conditions (**left**) and altered functions in lung cancer (**right**). Major regulators of cell cycle (Cyclins D and E, p53, 53BP1), proliferation (AKT), and chromatin accessibility (EZH2) interact with the lamin network to maintain cellular homeostasis and to coordinate structural integrity before and during cell division. In cancer, hijacking of these functions either by the mutational activation or the inactivation of proto-oncogenes and tumor suppressor genes contributes to the modulation and reprogramming of the lamin network, respectively. Specifically, aberrant AKT and cyclin D signaling in lung cancer induces B-type lamin depolymerization and enhanced proliferation, while the EZH2-mediated regulation of lamin A/C alters the transcriptional control of factors promoting cancer aggressiveness and EMT by down-regulating E-cadherin and upregulating vimentin. The arrows show up- and downregulation of the indicated factors altered by changes in lamin levels.

This review summarizes the functions of lamins in eukaryotic cells and focuses on the role of the lamin protein family in DNA damage response in lung cancer. We integrate current knowledge on lamin functions beyond acting as structural components of the cell nucleus (summarized in Figure 2). However, there is currently no clear picture emerging especially for the role of lamin B1 in lung cancer, which was reported to have either oncogenic or tumor-suppressive functions [67,71,72]. As lamin deregulation is commonly noted across cancer entities with sometimes conflicting observations, we also provide an overview of the currently available distribution of lamin A/C, B1, and B2 expression in diverse types of cancers, both in cell lines and patient-derived tissue (Table 1, Table 2 and Table 3). Notably, changes in lamin composition can also be found in many other tumor entities including the most frequent cancers of the breast [9,92], skin [55,93], and also digestive tract cancers [94,95,96,97,98]. On a general note, lamin expression is highly variable between cancers and different studies on lamins in large patient cohorts reported discrepancies concerning the clinical course and lamin expression (Table 1, Table 2 and Table 3). For instance, lamin A/C upregulation was reported in many tumor types such as prostate cancer, colorectal cancer, and glioblastoma, while lamin A/C was underrepresented in small-cell lung cancer, breast cancer, ovarian cancer, Ewing sarcoma, gastric cancer, and colon cancer (Table 1 and references therein). Patients with colorectal cancer (n = 656) expressing lamin A/C were reported to have a higher risk of disease progression than those without lamin A/C expression [95]. By contrast, loss of lamin A/C expression was associated with disease recurrence in another study involving colon cancer patients (n = 370). This discrepancy is also reflected in several studies, which reported different results when analyzing lamin A/C expression in NSCLC. Here, lamin A/C expression was altered in vitro in tumor cells compared to non-cancer cells and normal lung fibroblasts [68,69], whereas Jia et al. [67] found no differences in lamin A/C expression in a series of primary tumors by using immunohistochemistry. Moreover, different reports showed that either lamin B1 loss or lamin B1 overexpression promotes NSCLC aggressiveness [67,71,72]. Opposingly, lamin B2 overexpression unambiguously surfaced as an unfavorable event in lung cancer [73,74,75]. To sum this part up, the data availability concerning the biological function and the prognostic value of lamin expression is unsatisfactory except for lamin B2, which might serve as an indicator of aggressive disease courses.

To analyze if the conflicting results regarding lamin expression are due to different technical approaches, we inspected the methodology used in the respective studies (Tables S1–S3). These differences might arise when RNA levels and protein levels are compared or when technologies and platforms have limited overlap. However, methodological differences between studies could not fully explain the conflicting results. Additionally, the choice of reference groups (e.g., normal tissues, less-aggressive tumor types) did not correlate with the reported regulation of the lamins. However, as lamin isoform expression could be variable and differ between proliferating and resting cells, the ratios of lamin expression and correction for cell proliferation (e.g., by staining for Ki-67) would allow for a more precise view on the role of the differential expression of lamins across tumor types. Another important caveat has also recently been postulated by Roncato and colleagues [99], who showed that lamin A/C deficiency resulted in deformable nuclei, which enhanced migratory capacity but not metastasis in vivo. These results demonstrate that the readouts for evaluating functional consequences must be standardized when examining the complex interplay between nuclear membrane rigidity, migratory capacity, invasiveness, and metastatic ability. This also holds when lamins are analyzed in the context of resistance-associated EMT [70]. While lamins have a significant impact on the deformability of the cell nucleus and thus could favor the migration of potentially metastasizing cells, it is an oversimplification to assume that reduced A-type lamins automatically increase the metastatic ability of circulating cancer cells. Moreover, Nishikawa et al. [100] proposed an impact of distinct *LMNA* transcript variants on cancer cell proliferation. They focused on the *LMNA* transcript variant 6 (*LMNA-V6*), whose promotor region forms G-quadruplexes, thus increasing its transcriptional activity. Upregulation of *LMNA-V6* led to the downregulation of other lamin A transcripts. In addition, *LMNA-V6* expression also correlated with impaired p53 levels, suggesting a tumor-promoting function of this isoform [100]. Again, lamin B2 is the only lamin subtype whose expression is consistently annotated as pro-tumorigenic and correlates with poor patient prognosis across entities (Table 3 and Table S3). As for other cancer types, reports on lung cancer suggest that lamins are effectors of oncogenic processes that pave the way for malignant progression (Figure 2). However, a causal role for cancer development initiated by altered lamins was not established. This notion is also corroborated by the low frequency of lamin mutations in lung cancer and other diverse tumor types (Figure S1). Therefore, it remains to be determined if expression shifts or rather a specific combination of different lamin subtypes or transcript variants contribute to pro- or anti-tumorigenic properties of the lamin network. Data analyses of lamin subtypes should also consider transcript variants so as to dissect their impact on cancer development and progression. Moreover, the interaction between different lamins and their transcript variants that are governed by phosphorylation-dependent polymerization should be further investigated. Clearly, these ambiguous results call for a unified description of the methodology used in the manuscripts that deal with the role of lamins in cancer. Harmonization of protocols will then contribute to a deeper understanding of the role of lamins in cancer biology and progression.

**Table 1:** Lamin A/C expression levels in diverse types of cancer.

Cancer	Cell Line	Lamin Expression			Reference
		Regulation	Reference Parameter	Impact	
Ovarian Cancer	n = 1	↑*	Ovarian epithelial cells	/	[101]
Non-small cell lung cancer	n = 1 n = 1	↑ ↓	Non-cancer cells Normal lung fibroblast cells	/ /	[68] [69]
Small cell lung cancer	n = 3	↓	NSCLC cell lines	/	[66]
Osteosarcoma	n = 2	↓	Low aggressive cell line	Migration	[102]
**Cancer**	**Tumor** **Tissue**	**Lamin Expression**			**Reference**
		**Regulation**	**Reference Parameter**	**Impact**	
Prostate cancer	n = 46 n = 94 n = 94	↑* ↑* ↑	Benign sample High- vs. low-grade tissue /	/ Advanced stage Invasion	[103] [103] [104]
Glioblastoma multiforme	n = 152	↑	/	Poor prognosis	[105]
Colorectal cancer	n = 656	↑	/	Poor prognosis	[97]
Small cell lung cancer	n = 20 n = 33	↓ ↓	NSCLC NSCLC	/ /	[67] [66]
Breast cancer	n = 115	↓	Non-cancerous tissue	Poor prognosis	[9]
Ovarian cancer	n = 108	↓	Ovarian surface epithelial cells	/	[10]
Ewing Sarcoma	n = 64	↓	Metastatic vs. primary tumor	Poor prognosis	[106]
Gastric cancer	n = 52	↓	Normal tissue	Poor prognosis	[98]
Colon cancer	n = 35 n = 370	↓ ↓	Colonic mucosa No recurrence	/ Recurrence	[96] [95]

Overview of lamin A/C expression in tumor cell lines and tumor tissue as indicated. “Reference parameter” refers to the tissue or cell that served as a reference for lamin A/C expression. “n”: number of analyzed cells lines/tumor tissues; “↑”: increased expression; “↑*”: increased expression only for lamin A/C; “↓”: decreased expression.

**Table 2:** Lamin B1 expression levels in diverse types of cancer.

Cancer	Cell Line	Lamin Expression			Reference
		Regulation	Reference Parameter	Impact	
Ovarian cancer	n = 11	↑	Ovarian surface epithelial cells	/	[10]
Hepatocellular carcinoma	n = 5	↑	MIHA and HepG2	/	[107]
Melanoma	n = 6 n = 1 n = 2	↑ ↑* ↓*	Normal epidermal melanocytes / /	/ Migration Senescence	[55] [93] [55]
Prostate cancer	n = 1	↑*	/	Proliferation & invasion	[108]
Pancreatic cancer	n = 2	↓*	/	Migration & Invasion	[109]
**Cancer**	**Tumor** **Tissue**	**Lamin Expression**			**Reference**
		**Regulation**	**Reference Parameter**	**Impact**	
Ovarian cancer	n = 27	↑	Benign tumors	/	[110]
Pancreatic cancer	n = 5	↑	Normal pancreatic tissue	Poor prognosis	[109]
Hepatocellular carcinoma	n = 39 n = 364 + 229	↑ ↑	Cirrhosis and non-diseased tissue Low metastasis score group	/ Poor prognosis	[107] [111]
Clear cell renal cell carcinoma	n = 622	↑	Normal renal tissue	Poor prognosis	[112]
Non-small cell lung cancer	n = 483 n = 139	↑ ↓	Normal lung tissue Normal lung tissue	Poor prognosis Advanced stage	[71,72] [67]
Small cell lung cancer	n = 22	↓	Normal lung tissue	/	[67]
Breast cancer	n = 115	↓	Non-cancerous tissue	Poor prognosis	[9]
Colon cancer	n = 35	↓	Normal colonic mucosa	/	[96]

Overview of lamin B1 expression in tumor cell lines and tumor tissue as indicated. “Reference parameter” refers to the tissue or cell that served as a reference for lamin B1 expression. “n”: number of analyzed cells lines/tumor tissues; “↑”: increased expression; “↑*”: increased expression only for lamin A; “↓”: decreased expression. “↓*”: decreased expression only for lamin A.

**Table 3:** Lamin B2 expression levels in diverse types of cancers.

Cancer	Cell Line	Lamin Expression			Reference
		Regulation	Reference Parameter	Impact	
Colorectal cancer	n = 5	↑	Normal colon epithelial cell line	Proliferation	[94]
**Cancer**	**Tumor** **Tissue**	**Lamin Expression**			**Reference**
		**Regulation**	**Reference Parameter**	**Impact**	
Non-small cell lung cancer	n = 526 n = 20 n = 150 n = 135	↑ ↑ ↑ ↑	Normal lung sample Adjacent normal lung tissue Low-grade tumor Normal lung tissue	Poor prognosis / Poor prognosis Poor prognosis	[73] [74] [74] [75]
Breast cancer	n = 82 n = 1085	↑ ↑	Normal tissue Corresponding normal tissue	Poor prognosis /	[92] [92]
Ovarian cancer	n = 27	↑	Benign tumor	/	[110]
Colorectal cancer	n = 226	↑	Adjacent tissue	Poor prognosis	[94]

Overview of lamin B2 expression in tumor cell lines and tumor tissue as indicated. “Reference parameter” refers to the tissue or cell that served as a reference for lamin B2 expression. “n”: number of analyzed cells lines/tumor tissues; “↑”: increased expression; “↓”: decreased expression.

## 6. Conclusions

While lamins are indispensable for the structural integrity of the nucleus in normal cells, aberrant lamin subtype expression and composition as well as the lamin interactome in lung cancer and other tumor types is not yet fully understood. Even the role of the same lamins in and between cancer entities remains debatable. Nevertheless, it is evident that lamins orchestrate a variety of cellular cancer-related processes beyond their structural functions. In lung cancer, lamin composition and subtype expression govern the DNA damage response that is invoked by current therapies. The impact of lamins on therapy response and cancer progression deserves further investigation.

## Supplementary Materials

The following supporting information can be downloaded at: https://www.mdpi.com/article/10.3390/cancers15235501/s1, Figure S1: Mutation frequencies for *LMNA*, *LMNB1* and *LMNB2* genes across common cancer entities. Data were obtained from the “The Cancer Genome Atlas” (TCGA) projects available at the Genomic Data Commons Portal (https://portal.gdc.cancer.gov, accessed on 6 November 2023); Table S1: Methodological details for Table 1: Lamin A/C expression levels in diverse types of cancer, Table S2: Methodological details for Table 2: Lamin B1 expression levels in different types of cancer, Table S3: Methodological details for Table 3: Lamin B2 expression levels in different types of cancers.
