# Supplementary material for: The Multifaceted Roles of Lamins in Lung Cancer and DNA Damage Response
Source: Cancers (Basel). 2023 Nov 21;15(23):5501. doi: 10.3390/cancers15235501 (PMC10705174; doi:10.3390/cancers15235501)
Supplement: Supplementary file 1 [file cancers-15-05501-s001.zip › cancers-2466657-supplementary.pdf]

Supplementary Materials: The Multifaceted Roles of Lamins in Lung Cancer and DNA Damage Response

Janina Janetzko, Sebastian Oeck * and Alexander Schramm

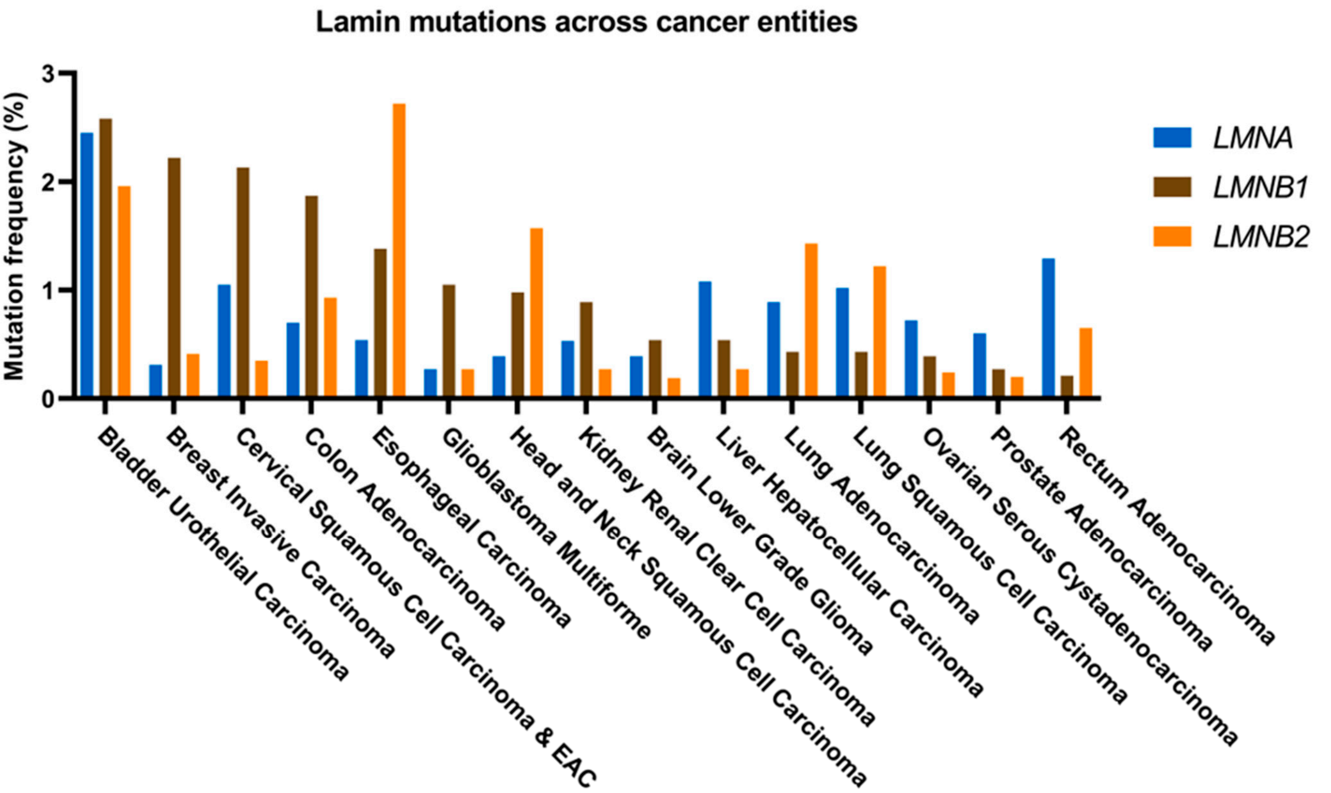

Figure S1. Mutation frequencies for *LMNA*, *LMNB1* and *LMNB2* genes across common cancer entities. Data were obtained from the "The Cancer Genome Atlas" (TCGA) projects available at the Genomic Data Commons Portal (https://portal.gdc.cancer.gov).

Table S1. Methodological details for Table 1: Lamin A/C expression levels in diverse types of cancer.

Cancer	Cell line	Method	Technical details	Reference
Ovarian Cancer	n = 1	IF	Sigma Aldrich (L1293) 1:100	[101]
Non-small cell lung cancer	n = 1	IF & WB	Cell Signaling Technology 1:2000 (WB)	[68]
	n = 1	LC-MS/MS	/	[69]
Small cell lung cancer	n = 3	RT-seq	mRNA	[66]
Osteosarcoma	n = 2	IF	Santa Cruz Biotechnology	[102]
**Cancer**	**Tumor tissue**	**Method**	**Technical details**	**Reference**
Prostate cancer	n = 46	MS	/	[103]
	n = 94	IHC	Abcam (133A2) 1:150	[103]
	n = 94	IHC	Sigma Aldrich (HPA006660) 1:10	[104]
Glioblastoma multiforme	n = 152	RNA-seq	mRNA	[105]
Colorectal cancer	n = 656	IHC	Abcam (Jol2) 1:10	[97]
Small cell lung cancer	n = 20	IHC	Santa Cruz Biotechnology (N-18) 1:100	[67]
	n = 33	IHC	Cell Signaling Technology 1:50	[66]
Breast cancer	n = 115	RT-qPCR	mRNA	[9]
Ovarian cancer	n = 108	IHC	/	[10]
Ewing Sarcoma	n = 64	RNA	/	[106]
Gastric cancer	n = 52	RT-PCR	mRNA	[98]
Colon cancer	n = 35	IHC	Own-designed antibody 1:300	[96]
	n = 370	IHC	Abcam (Jol2)	[95]

"n": number of analyzed cells lines/tumor tissues; "IF": Immunofluorescence; "IHC": Immunohistochemistry; "LC-MS/MS": Liquid chromatography with tandem mass spectrometry; "RT-qPCR": Real time quantitative polymerase chain reaction; "WB": Western blot.

Table S2. Methodological details for Table 2: Lamin B1 expression levels in different types of cancer.

Cancer	Cell line	Method	Technical details	Reference
Ovarian cancer	n = 11	Northern Blot	Total RNA	[10]
Hepatocellular carcinoma	n = 5	WB	Chemicon 1:300	[107]
Melanoma	n = 6	RNA-seq	HiSeq with paired-end	[55]
	n = 1	Overexpression	GFP-fused lamin B1	[93]
	n = 2	Knockdown	siRNA	[55]
Prostate cancer	n = 1	Overexpression	Lentiviral vector pLAS2.Pneo	[108]
Pancreatic cancer	n = 2	Knockdown	siRNA	[109]
**Cancer**	**Tumor tissue**	**Method**	**Technical details**	**Reference**
Ovarian cancer	n = 27	LC-MS/MS	/	[110]
Pancreatic cancer	n = 5	WB & RT-PCR	Santa Cruz Biotechnology (sc-6216) & RNA	[109]
Hepatocellular carcinoma	n = 39	2-DE, MS/MS	/	[107]
	n = 364 + 229	RNA	mRNA	[111]
Clear cell renal cell carcinoma	n = 622	IHC	Abcam (ab16048) 1:1000	[112]
Non-small cell lung cancer	n = 483	IHC	Sigma-Aldrich & Santa Cruz 1:100	[71,72]
	n = 139	RNA	mRNA	[67]
Small cell lung cancer	n = 22	IHC	Sigma-Aldrich & Santa Cruz 1:100	[67]
Breast cancer	n = 115	RT-qPCR	mRNA	[9]
Colon cancer	n = 35	IHC	Own-designed primary antibody 1:300	[96]

"n": number of analyzed cells lines/tumor tissues; "IHC": Immunohistochemistry; "LC-MS/MS": Liquid chromatography with tandem mass spectrometry; "RT-qPCR": Real time quantitative polymerase chain reaction; "WB": Western blot; "2-DE": 2-dimensional gel electrophoresis.

Table S3. Methodological details for Table 3: Lamin B2 expression levels in different types of cancers.

Cancer	Cell line	Method	Technical details	Reference
Colorectal cancer	n = 5	WB	Abcam (151735)	[94]
**Cancer**	**Tumor tissue**	**Method**	**Technical details**	**Reference**
Non-small cell lung cancer	n = 526	/	/	[73]
	n = 20	qPCR	/	[74]
	n = 150	IHC	mRNA	[74]
	n = 135	IHC	Abcam	[75]
Breast cancer	n = 82	IHC	Abcam (ab151735) 1:100	[92]
	n = 1085	IHC	/	[92]
Ovarian cancer	n = 27	LC-MS/MS	/	[110]
Colorectal cancer	n = 226	IHC	Abcam (ab151735) 1:100	[94]

"n": number of analyzed cells lines/tumor tissues; "IHC": Immunohistochemistry; "LC-MS/MS": Liquid chromatography with tandem mass spectrometry; "qPCR": quantitativepolymerase chain reaction; "WB": Western blot.
